# Attention-Deficit Hyperactivity Disorder (ADHD) and narrative discourse in older adults

**DOI:** 10.1590/1980-57642018dn12-040006

**Published:** 2018

**Authors:** Rafael Coelho, Paulo Mattos, Rosemary Tannock

**Affiliations:** 1 Instituto D’Or de Pesquisa e Ensino (IDOR), Rio de Janeiro, RJ, Brazil; 2 Instituto de Psiquiatria (IPUB), Universidade Federal do Rio de Janeiro (UFRJ), Rio de Janeiro, RJ, Brazil; 3 The Hospital for Sick Children (SickKids), Toronto, ON, Canada; 4 Ontario Institute of Studies in Education, University of Toronto, Toronto, ON, Canada.

**Keywords:** ADHD, language, narrative discourse, communication disorder, older adults, aging, TDAH, linguagem, discurso narrativo, transtorno de comunicação, adultos mais velhos, envelhecimento

## Abstract

Attention-Deficit Hyperactivity Disorder (ADHD) encompasses other symptoms besides inattention, hyperactivity, and impulsivity, such as language problems. ADHD can have a non-remitting course and is also found in older individuals, although there are no studies on language problems in elderly individuals with the disorder.

**Objective:**

To investigate the presence of language impairment in older adults with ADHD.

**Methods:**

Language impairment was investigated in three older ADHD adults, and compared with two matched control subjects using a narrative discourse task. The transcript discourses were evaluated based on the Trabasso Model for discourse analysis, and then processed by the Speech Graph Analysis software.

**Results:**

Compared to control subjects, ADHD patient discourse had more Plot components and their networks exhibited more Edges. The patients had higher scores on the Narrative Inefficiency, Density and Diameter Indexes as well as on the Average Clustering Coefficient. The networks of control subjects were sequential, with little or no recursiveness, whereas those of ADHD subjects were convoluted.

**Conclusion:**

Our results suggest that language deficits described in children, adolescents and young adults with ADHD may persist in older adults with the disorder.

## RESUMO

O Transtorno do Déficit de Atenção/Hiperatividade (TDAH) compreende diversos outros sintomas além de desatenção, hiperatividade e impulsividade, tais como problemas de linguagem. O TDAH pode cursar sem remissão, tendo sido demonstrado em indivíduos mais velhos, porém não há estudos investigando a linguagem em idosos com o transtorno.

**Objetivo:**

Investigar a presença de déficits de linguagem em adultos mais velhos portadores de TDAH.

**Métodos:**

Avaliou-se a presença de comprometimento de linguagem em três adultos mais velhos com TDAH, que foram comparados a dois indivíduos controles pareados, através de tarefa de discurso narrativo. A transcrição das narrativas foi avaliada com base no modelo de análise de narrativa de Trabasso, e então analisada com uso do programa *Speech Graph Analysis*.

**Resultados:**

Comparados aos indivíduos controles, adultos mais velhos com TDAH utilizaram mais componentes (Núcleos) da história em suas narrativas e suas redes mais Arestas. Eles apresentaram escores mais altos no Índice de Ineficiência, Diâmetro e Densidade e também no Coeficiente Médio de Grupamento. As redes dos indivíduos controles foram lineares e sequenciais, com pouca ou nenhuma recursividade, ao contrário das redes de adultos com TDAH.

**Conclusão:**

Estes resultados sugerem que problemas de linguagem já descritos em crianças, adolescentes e adultos com TDAH, podem persistir em adultos mais velhos com o transtorno.

It is now well established that Attention-deficit hyperactivity disorder (ADHD) comprises more deficits than its defining symptoms of inattention, hyperactivity, and impulsivity; a wide range of neuropsychological deficits have also been described in ADHD.^1^ Among those deficits, a growing body of evidence has demonstrated language impairment in ADHD, such as deficits in expressive language (sentence initiation, word articulation and speech organization) and more frequent inappropriate pragmatic behavior (no response to a question or request, more interruption/overlap of speakers, no feedback to speakers, poor cohesion, and lack of subject specificity).^2^^-^ 4 Some studies have demonstrated linguistic deficits somewhat similar to those seen in Autism Spectrum Disorders.^5^ Although ADHD was first thought to be a disorder present only in childhood and adolescence; it is now recognized as a persistent disorder not only into adulthood but also older age.^6^

Here we present a case series in which we demonstrate language impairment in three older adults with ADHD using a novel software program to analyze macrostructural aspects of language with the Trabasso coding approach for narrative discourse analysis.^7^^-^ 9 Discourse is a complex linguistic activity comprising different levels (phonological, morphosyntactic, semantic-lexical, as well as semantic-pragmatic) in conjunction with other cognitive domains, such as executive functions.^10^ Narrative discourse, unlike other types of discourse – such as spontaneous and descriptive discourse – requires the speaker to verbally recount an episode experienced in the present (perception of visual stimuli, usually scenes) or past (memory) while respecting the temporal, causal and spatial relationships among events that unfold in particular scenarios, offering an ecological advantage in language evaluations.^10^

Although *overall* cognitive functioning in older adults with ADHD is not usually impaired when compared to age-matched individuals,^11^ there are no studies investigating narrative language abilities in this group.

## METHODS

### Sample

The three patients reported were referred for neuropsychological examination at our private clinic for memory disorders (a 64-year-old male, merchant; a 61-year-old female nurse; and a 59-year-old female math teacher). All patients signed an informed consent approved by the Ethics Committee. The patients presented problems involving memory functioning (retaining and recalling new information). All patients described a history of inattention symptoms dating back to early adolescence (before the age of 12), lifelong problems with daily life management (suggestive of impaired executive functions) and persistent forgetfulness, which they considered to be worse now than before. The patients had never received a psychiatric diagnosis and were drug-naïve. None of the patients had impairment in their Activities of Daily Living (ADL) according to reliable collateral reports. All patients underwent the following evaluation: a) psychiatric, b) neurological, c) neuropsychological assessment, and d) neuroimaging. Neuropsychological testing comprised the following: a) auditory verbal memory (Logical Memory from the WAIS-IV Battery and Rey Auditory Verbal Learning Test), b) visual memory (Benton Visual Memory Test), c) working memory (Digit Span and Visual Memory Span), d) attention (Digit Span and Five Digits Test), e) visuoconstructive skills (Block Design), f) executive functions (Hayling Test, Word Association (FAS), Five Digits Test, Trail Making B), and g) a language evaluation (Naming, Word Association (FAS), Reading and Writing). The results from all patients were within normal range, except for the Trail Making B test of the male patient, but this result had no clinical correlate. The narrative discourse analysis was performed on a separate occasion. All MRI scan results were considered normal by a trained neuroradiologist.

Patients reported no depressive symptomatology, and their scores on the Geriatric Depression Scale (GDS) were within normal range. None of the patients displayed any primary language deficit suggestive of Primary Progressive Aphasia (PPA). Also, they did not exhibit impaired performance on any of the cognitive domains in the neuropsychological evaluation; therefore, they did not fulfill DSM-5 diagnosis for Major Neurocognitive Disorder (dementia) or Mild Neurocognitive Disorder (MCI – Mild Cognitive Impairment). All patients received an ADHD diagnosis according to guidelines proposed by Kooij.^12^ The control subjects were a 63-year-old-woman, merchant and a 62-year-old-woman school teacher. Both controls had an active working life at the time of evaluation; had no memory or attention complaints, and neither had any impairment in Activities of Daily Living (ADL) according to reliable collateral reports. The patients and control individuals were matched by the Global Assessment Functioning (GAF) scale score, educational level and age.

### Narrative task

Narrative discourse can be distinguished from other types of discourse (such as spontaneous and descriptive) because it requires the individual to verbally reproduce a story while respecting the temporal and causal relationships among events. For this, we used visual stimuli from the book “Frog, Where are you?”^13^ which has been used previously for children with ADHD.^4^^,^ 14 This book, containing no words, tells the story of a boy who loses his pet frog and begins a journey to get it back. The story unfolds in twenty-four frames evenly distributed throughout the book pages. These 24 drawings show events (narrative components) comprising a *main plotline* (the search for the pet frog and the events directly related to the aim of getting it back) and a *secondary plotline* (peripheral series of events which are not necessary for good comprehension of the story). There are many advantages regarding this strategy: a) a book containing no words with a coherent story provides an ecological way of eliciting and accessing narrative discourse; b) the events (in both main and secondary plotlines) happen sequentially one at a time, allowing investigation of the flow of the narrative discourse; c) each event occurs just once in the story, so linearity is expected in narratives; d) the book has been previously used in many studies as visual stimuli for narrative analysis^4^^,^ 9 ^,^ 14 in five different languages with a wide range of ages, including children, adolescents and adults.^7^^,^ 9 ^,^ 15

The wordless picture book is presented to the patient who is given the following instruction: “Here is a picture book. It’s about a boy and his pets, a frog and a dog. You should look at each page, and then tell me the story. I’m going to record the story you produce. You can look through the whole book as many times as you want before we start. It is not necessary to memorize the book, you’ll keep it with you. You should try telling the best possible story, pretending that I don’t know it.”

The narrative produced by the patient is first recorded, then transcribed and submitted for analysis as described below.

### Narrative evaluation and graph measures

We used the previous studies of Slobin, Trabasso and Von der Broek, Stein and Gleinn, Nickels and Trabasso and Stein^9^^,^ 16 ^-^ 18 , which propose complementary methods to analyze macro and microstructural aspects of narratives (in this case series we focused on the macrostructural aspects only). Stories are decomposed into *primary events* (from the main plotline – the events directly related to the main goal of finding the frog and getting it back), and *secondary events* of the narrative (from the secondary plotline). The narrative is then transformed into a written sequence in order to be analyzed by software. We used the Speech Graphs Analysis (SGA) software program^19^^,^ 20 for the narrative discourse transcripted and analyzed by the Trabasso Model of discourse analysis^16^^-^ 18 ^,^ 21 ^,^ 22 generating a written sequence of the story´s components. The output is a word-graph^23^ representing each component of the story as a *node*, and the link between the story components as an *edge*.

The *Speech Graphs Analysis* (SGA- http://neuro.ufrn.br/softwares/speechgraphs)^19^^,^ 20 ^,^ 24 is an open source software program that portrays a text (in this case the transcribed narrative as a graph), representing each narrative nucleus as a node, and the link between each as an edge. The SGA quantifies the graph variations to calculate 14 speech graph attributes comprising general attributes, thereby providing several different measures, described in detail elsewhere.^19^ In this case series, we focused on five parameters: a) WC – Word Count – total number of components from the final sequence (repeated components are included), in our study the Number of Plot Component; b) Nodes – total number of different narrative components produced (main elements of the story), in our study the Number of Distinct Plot Component; c) Edges – the links between narrative components; c) Density – index reflecting linearity of the narrative, in this case a measure of narrative efficiency ; d) Diameter – representing the maximum amount of linear information; also a measure of narrative efficiency; e) CC – Average Clustering Coefficient - representing major repeated nodes in the network; this is used as a measure of an inefficient narrative. Since the book has no repeated events, a zero value is expected for CC. Positive values indicate inefficient or disorganized narratives.

For comparison, we show the graphs of matched control individuals without memory complaints and normal performance on Activities of Daily Living; they belonged to the same social class as the patients and were invited to participate by the research group. The quantitative data displayed here aims to show how specific parameters can be used to investigate difference between groups; the small sample size precluded statistical analysis, i.e., comparisons between groups.

## RESULTS

**Figure 1:**
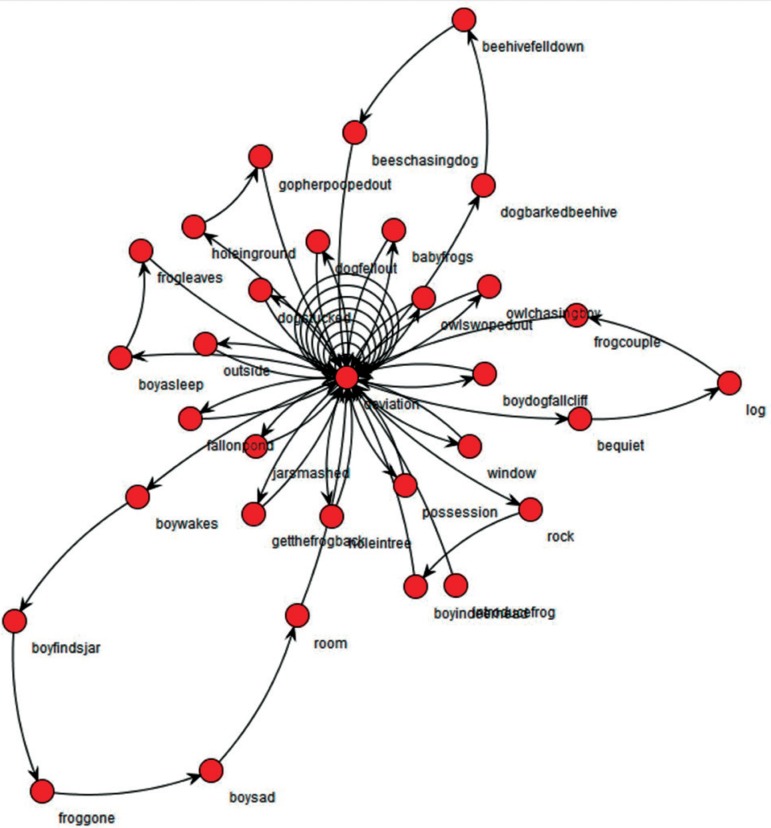
ADHD subject (patient 3)

**Figure 2:**
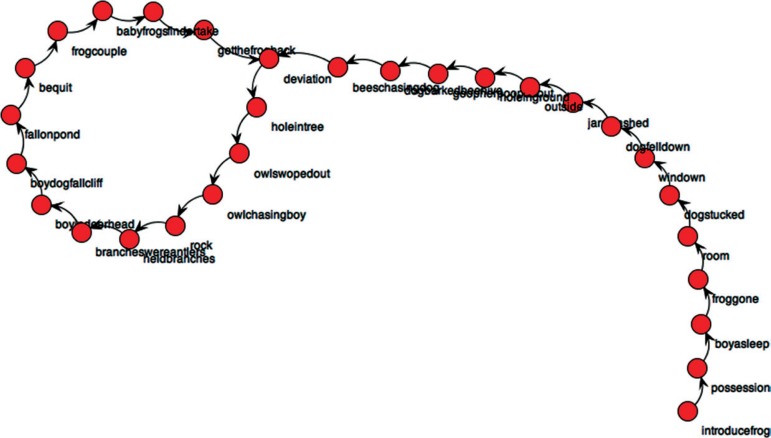
Control Subject (control 2)

The graphs produced by SGA analysis from the narrative discourse of an ADHD patient, and one graph of a control individual are shown below (Figures 1 and 2); the first portrays a clear lack of linearity, unlike the second. In addition, the graph for the ADHD patient has a significantly greater density. Graphs of all ADHD patients and control individuals are shown in the supplementary section (available on www.demneuropsy.com.br/).

**Figure 3:**
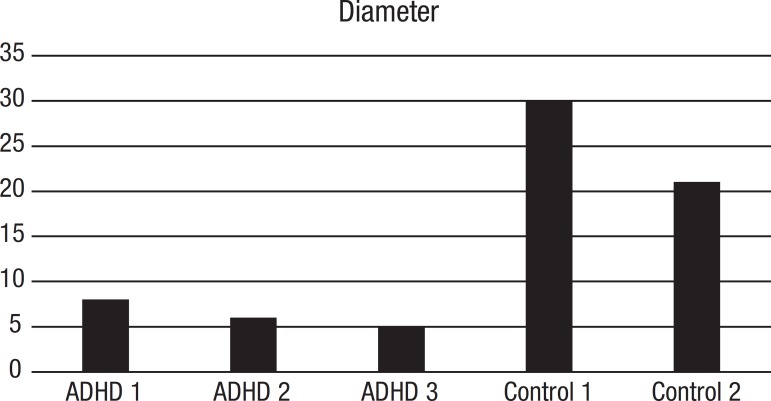
Diameter is the length among the longest of the shortest path between the node pairs of a network. Diameter indicates the linearity of the narrative

**Figure 4:**
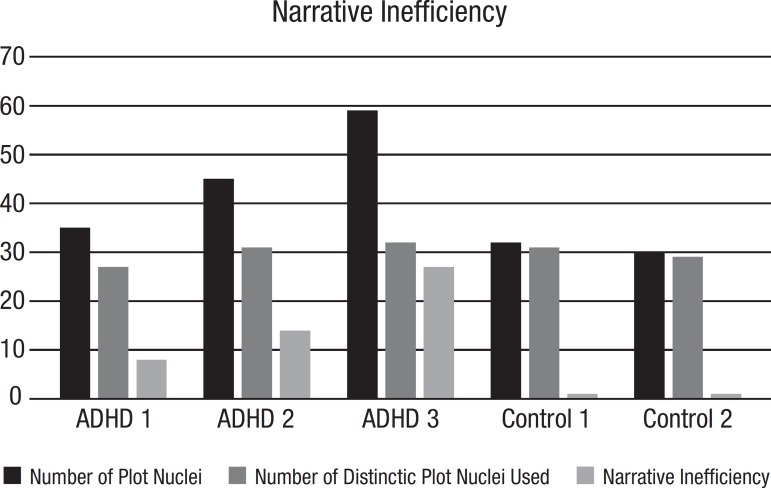
Narrative Inefficiency. ADHD patients used more plot nuclei than control individuals without improvement in their narratives

**Figure 5:**
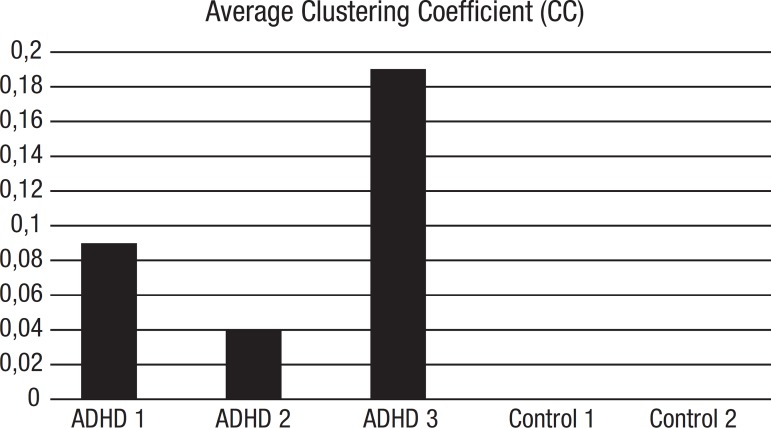
Average Clustering Coefficient (CC): Given a node n, the Clustering Coefficient Map (CCMap) is the set of fractions of all n neighbors that are also neighbors of each other. Average CC is the sum of the Clustering Coefficients of all nodes in the CCMap divided by number of elements in the CCMap. CC indicates convoluted or disorganized narratives

Table 1 shows the graphs with specific parameters for patients and controls. Of all parameters provided by the software, Diameter, Density and CC seem to be those which differ the most between controls and ADHD; data are shown in Figures 3, 4 and 5.

**Table 1:** SGA Parameters of graphs for ADHD subjects and controls.

	Number of plot components	Number of distinct plot components used	Narrative inefficiency index	Edges	Density	Diameter	Average clustering coefficient
Patient 1	35	27	8	34	0.09	8	0.09
Patient 2	45	31	14	44	0.08	6	0.04
Patient 3	59	32	27	58	0.07	5	0.19
Control 1	32	31	1	31	0.06	30	0
Control 2	30	29	1	29	0.07	21	0

## DISCUSSION

At the overall macrostructural level – i.e. from the visual inspection of the graphs - there is a clear contrast between the narratives of older adults with and without ADHD. While control narratives were typically sequential, with no or little recursiveness (as expected given the linearity of events in the book), narratives were convoluted in ADHD patients. Clinically, their discourse was poorly organized, redundant, often portraying a more descriptive than narrative discourse, with too many details and some embellishment. This last aspect can be quantitatively demonstrated by the *Diameter* of narrative discourses, as the *Diameter* is the length of the longest among the shortest path between the node pairs of a network, and here indicates the linearity of the narrative (Figure 3). Although the *Number of Plot Components* (network *nodes)* was similar, two ADHD individuals clearly provided more narrative nuclei (as indicated by the *Number of Plot Nuclei*) when compared to controls, indicating the unnecessary use of more narrative components or nuclei in their narratives (*Narrative Inefficiency,* Figure 4) without any improvement in the discourse, or even jeopardizing its quality.

Since the *Average Coefficient Cluster* (CC) indicates the occurrence of repeated nodes in the network and there are no repeated events in the book, positive non-zero CC values in all ADHD patients indicate inefficient or disorganized narratives (Figure 5). In summary, the narrative discourses of the patients differed from those of control individuals.

The ADHD patients were thoroughly investigated through clinical, neuropsychological and neuroimaging evaluation and we are therefore confident that their language deficits were unrelated to abnormal aging, albeit Mild Cognitive Impairment or Primary Progressive Aphasia. These results suggest that language deficits previously described in children, adolescents and adults with ADHD may persist into old age. Notably, the new methodology used in this study provides measurable results for language deficits previously described qualitatively.

The small sample size precluded statistical comparisons; these preliminary results aim to demonstrate that quantitative analysis with specific scores can be useful in the investigation of language impairment in ADHD. Although individuals were not assessed by blinded examiners, their discourses were transcribed and analyzed by the software, making potential bias negligible.

In conclusion, similar to that demonstrated in younger individuals with ADHD, our results suggest that language impairment is also present in elderly patients. Evaluations based on a more complex and quantitative analysis might provide a deeper understanding of the impact of ADHD on cognition. From a clinical perspective, language impairment can be objectively described to patients and may also be used to address treatment strategy efficacy. Further studies assessing narrative discourse involving a larger number of ADHD adults are warranted.
